# A Comprehensive Physiotherapeutic Intervention for Complex Proximal Tibia Fracture With Ilizarov Fixator and Foot Drop in 18-Year-Old Adult: A Case Report

**DOI:** 10.7759/cureus.58355

**Published:** 2024-04-15

**Authors:** Anam F Pathan, Subrat Samal

**Affiliations:** 1 Musculoskeletal Physiotherapy, Ravi Nair Physiotherapy College, Datta Meghe Institute of Higher Education and Research, Wardha, IND

**Keywords:** activity of daily living (adl), physiotherapy rehabilitation, foot drop, ilizarov fixators, proximal tibia fracture

## Abstract

The susceptibility of the tibia to fractures arises from its exposed position, making it a commonly affected area. The proximal tibia exhibits a wide metaphyseal region that gradually narrows distally, forming a triangular shape. The extended tibia shaft articulates with the fibula, talus, and distal femur. We have discussed the case of an 18-year-old male who experienced a road traffic accident on January 7, 2023, involving a collision between his bike and four-wheeler, resulting in high-energy forces impacting his left lower limb. As a consequence, he lost mobility in the left lower limb. Upon examination, he was diagnosed with a compound grade 3C proximal tibia fracture treated with Ilizarov fixators, accompanied by a neurovascular deficit leading to a foot drop on the left side. Additionally, he had a previous operative case involving a femur shaft fracture on the left side, which was managed with in situ implants. It concluded that the rehabilitation approach was effective in pain reduction, improving range of motion, muscle strength, and reducing sensory impairment. Improved results on the lower extremity functional scale and the foot and ankle ability measures showed that the physiotherapy method had been successful in helping the patient regain independence in everyday activities. The success of rehabilitation and the recovery of patients are greatly influenced by post-operative physical therapy.

## Introduction

A triangle is formed by the proximal tibia's wide metaphyseal region narrowing distally. The distal femur, talus, and fibula articulate with the long tibia shaft [1]. A tibia fracture can occur at any age and takes many different shapes. A brace or plaster cast can be used to treat simple fractures with modest soft-tissue injuries, while plastic surgery, osteosynthesis, or even amputation is required for more complex fractures [2]. The AO/OTA's classification plan for tibial fractures based on afflicted bone sections states that type 1 tibial fractures are proximal, type 2 diaphyseal fractures are diaphyseal, and type 3 tibial fractures are distal. Proximal tibial fractures affect part of the bone called the proximal metaphyseal region; they may migrate into the adjacent knee joint. The classification of proximal tibial fractures by AO/OTA includes type 1 A, which is considered extra-articular; type 1 B, which is considered partial intra-articular; and type C, which is considered full intra-articular. Furthermore, these fractures may be either closed or open [3]. Because of the surrounding soft-tissue damage and the potential for ambient pollutants to enter the fracture site and communicate with it, open fractures are often linked to high-energy trauma and carry an increased risk of infection [4]. Kim et al. further divided these high-energy open fractures into A, B, and C categories based on the degree of soft tissue damage, the requirement for vascular reconstruction, and the decreasing prognosis. Type 3A refers to high-energy trauma, independent of the size of the incision, or open fractures with sufficient soft tissue coverage of a broken bone despite severe soft tissue laceration or flaps; type 3B refers to open fractures with periosteal stripping, bone exposure, and significant soft tissue damage loss. This is typically linked to severe pollution, and type 3C open fractures linked to vascular damage that need to be repaired [5]. The tibia is prone to fractures because of its exposed position, which makes it a common injury target. An estimated 8.1 to 37.0 tibia fractures occur for every 100,000 people [6].

All tibial shaft fractures, which frequently come from forceful trauma, and extra-articular proximal tibial fractures make up between 5 and 11 percent. They cause severe wounds that affect the surrounding soft tissues and bones [7]. Over 80% of the lower extremity's weight-bearing stress is carried by the tibia, a long bone along a triangular cross-section [8]. There is controversy surrounding the treatment of open tibial diaphyseal fractures that result in bone loss. Treatment options include grafting with or without plastic reconstruction, nailing, ring fixators, and external fixators [9]. A novel kind of circular or ring external fixator was first introduced in the 1950s by Russian professor Gavriil Abramovich Ilizarov (1921-1992). The more challenging cases of infection non-union, limb shortening by bone lengthening, and joint and bone deformity repair, either alone or in combination, are treated using ring external fixators [10]. Among the most popular surgical procedures for open tibia fractures is the external fixation approach, yet it is frequently linked to problems such as pin tract infection, fixation loosening, non-union, and malunion of the fracture. Because of this, while treating open tibia fractures, surgeons may use internal fixation (IMN) [11].

## Case presentation

Patient information

We present the case of an 18-year-old male who met with a road traffic accident on January 7, 2023, resulting in a collision between a bike and a four-wheeler after which he was unable to move the left lower limb. The patient was taken to a nearby local hospital, where first aid was done, and referred to Acharya Vinobha Bhave Rural Hospital, where an investigation like an x-ray was done. Based on an x-ray investigation, the patient was diagnosed with a midshaft femur fracture and compound grade 3 C fracture of the proximal tibia of the left side. The patient was operated on in January 2023 for a midshaft femur fracture with open reduction, internal fixation of the intramedullary rod, and nailing in the left leg. On February 2023, the patient was operated on for a compound grade 3C fracture of the proximal tibia with the application of extracutaneous plating over the distal femur and proximal tibia. Post-operatively, one month later, the patient was able to walk with a walker and has been discharged. The patient came to the hospital in April 2023 with complaints of pain and the discharge of pus from the site of extracutaneous plating over the left leg. Removal of extracutaneous plating and application of Ilizarov ring fixators on the left tibia were done on April 12, 2023. After six months, the patient came to the hospital with complaints of heaviness and pain with discharge of blood from the previous scar on the lower end of the left thigh. On consulting with orthopedics, he was diagnosed as an old operated case of compound grade 3c proximal tibia fracture with Ilizarov fixators and neurovascular deficit foot drop left side, and an old operated case of shaft of femur fracture with implants in-situ left side. On November 22, 2023, the patient was operated on for bone grafting of the shaft of the femur on the left side. Now patients have been referred to physiotherapy rehabilitation for post-operative pain, reduced muscle strength, decreased range of motion (ROM), and sensory impairment in the left foot. Table 1 summarizes the timeline of the events.

**Table 1:** Timeline of events RTA: road traffic accident; ORIF: open reduction and internal fixation.

Events	Dates
RTA of bike collision	07 January 2023
Patient admitted to hospital	08 January 2023
Operated with ORIF of intramedullary rod and nailing for midshaft femur fracture	08 January 2023
Operated with extracutaneous plating over the distal femur and proximal tibia	February 2023
Removal of extracutaneous plating and application of Ilizarov ring fixators on the left tibia	12 April 2023
Patient was operated on for bone grafting of the shaft of the femur on the left side	22 November 2023
Post-operative physiotherapy started on	24 November 2023

**Figure 1:**
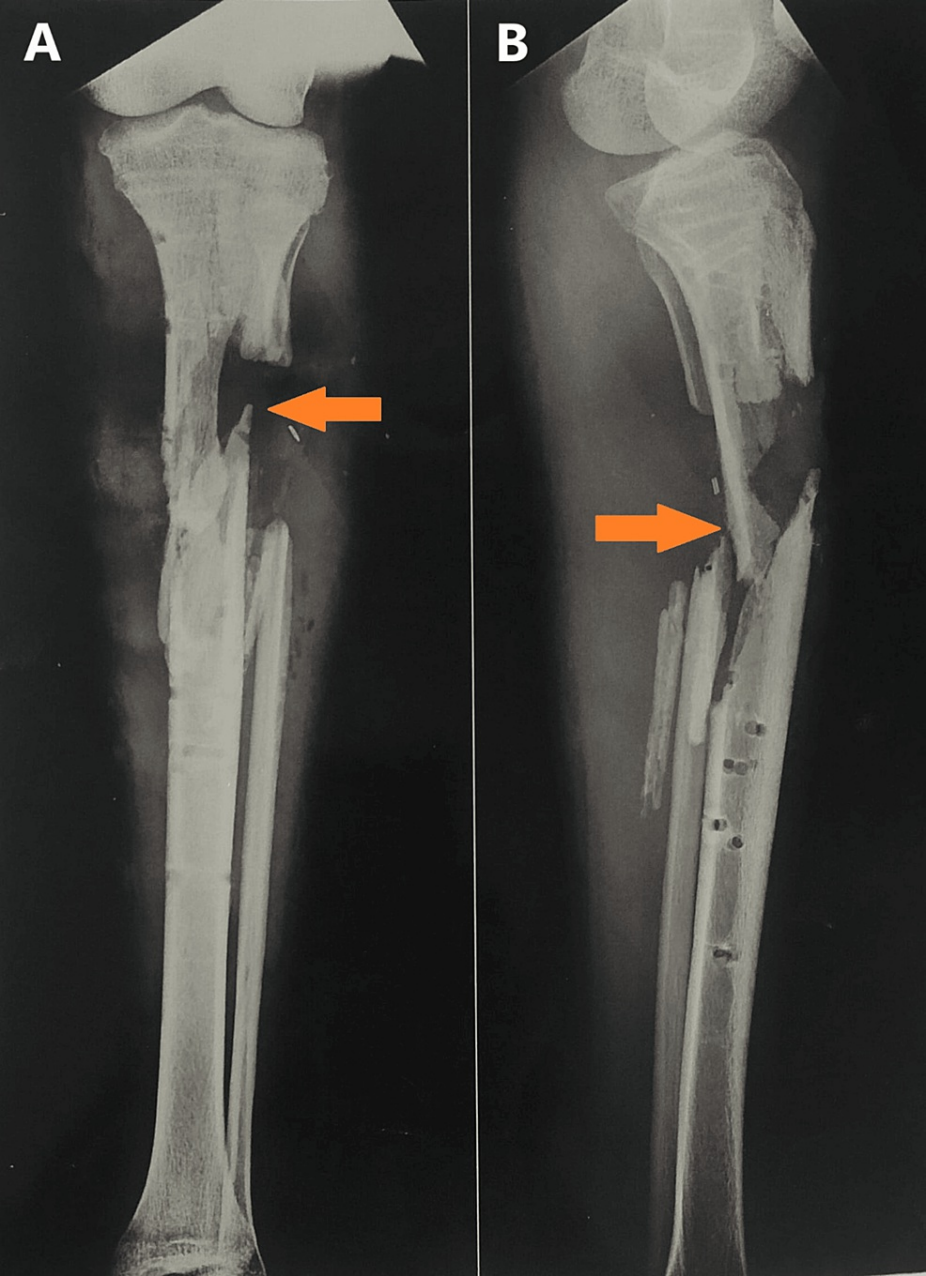
Pre-operative x-ray in anterior and lateral view of compound grade 3c proximal tibia fracture of left lower limb. A. Orange arrow shows a pre-operative x-ray in anterior view of a compound grade 3c proximal tibia fracture of the left lower limb. B. Orange arrow shows a pre-operative x-ray in lateral view of a compound grade 3c proximal tibia fracture of the left lower limb.

**Figure 2:**
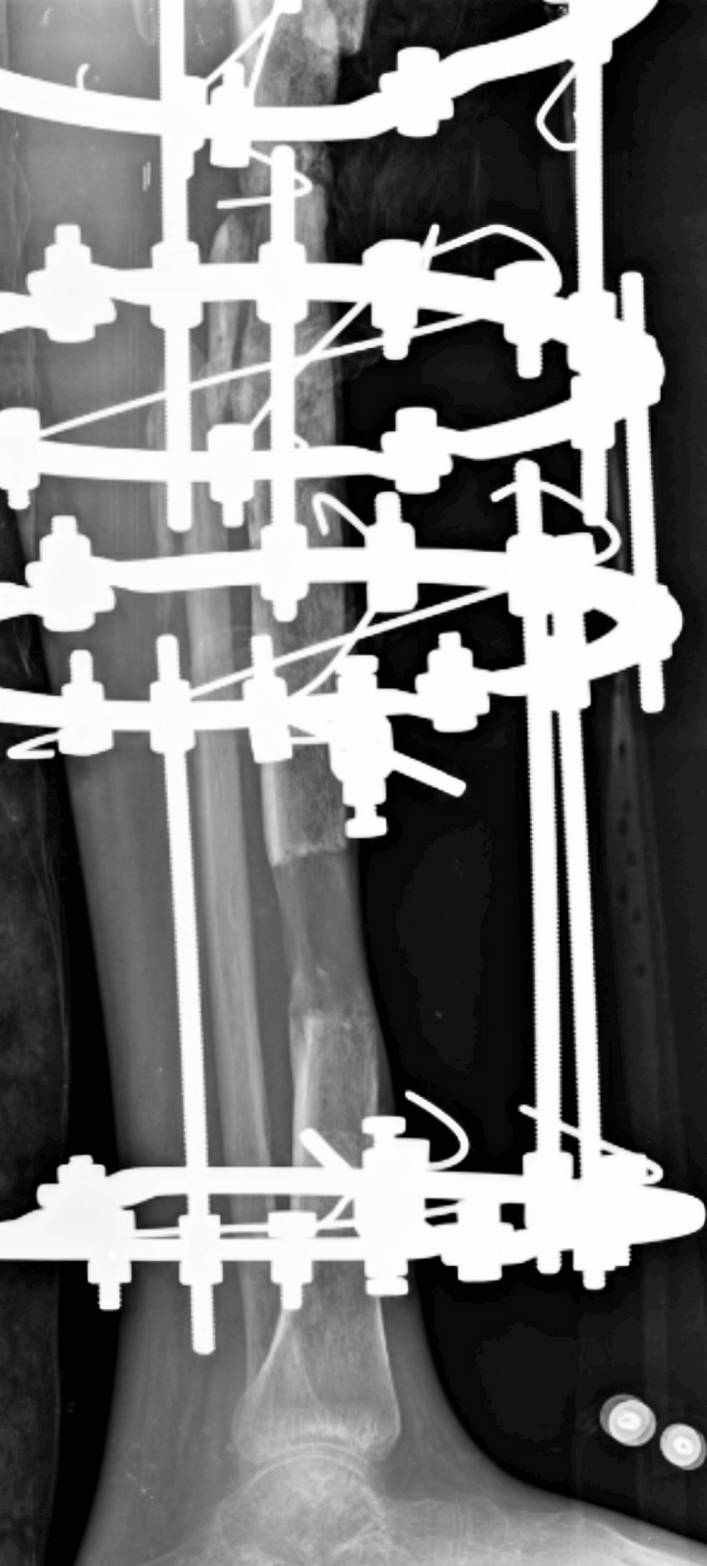
Post-operative x-ray of a compound grade 3c tibial fracture with Ilizarov fixators.

A radiographic examination of the affected extremity was obtained pre-operatively, as shown in Figure 1, which revealed a compound grade 3c proximal tibia fracture after the removal of extracutaneous plating. Another radiographic was obtained after stabilizing the tibia with Ilizarov ring fixators. The application of Ilizarov ring fixators was done on April 12, 2023. In Figure 2, the gap is shown in an anterior and lateral view after harvesting bone from the tibia for bone grafting of the shaft of the femur of the left side post-operatively on November 22, 2023. 

Clinical findings

**Figure 3:**
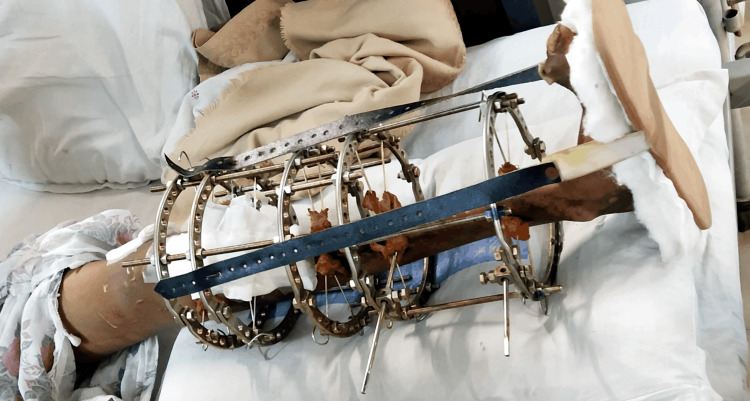
Ilizarov fixators with foot drop splints in the left lower limb.

After giving consent, the patient underwent an examination. The patient was assessed in a long sitting position with the operated limb elevated on a pillow, the hip and knee slightly flexed (around 15 degrees), and the ankle in a plantar flexion position. The examination revealed the presence of an Ilizarov ring fixator with a foot drop splint on the left leg, as shown below in Figure 3. Local examination of the left lower limb indicated a 5 cm scar from previous surgery on the lateral aspect of the knee joint. Palpation revealed grade 1 tenderness (patient complaints of pain) on the left shin of the tibia. Muscle wasting in the left thigh was evident compared to the right thigh by measuring with an inch tape 10 cm above the knee. The affected left lower extremity exhibited a restricted range of motion (ROM), whereas the unaffected extremities demonstrated normal ROM measured with a goniometer, as detailed below in Table 2. The pre-intervention muscle strength grading for quadriceps femoris and hamstrings on the left side was weak and painful during resisted isometric contraction (RIC). Post-intervention, the strength improved strong and painless. Table 3 presents the results of manual muscle testing (MMT) for the lower limb. Sensory examination indicated diminished light touch and pinprick sensations over the dorsum and sole of the left foot.

**Table 2:** Range of motion examination. ROM: range of motion; NA: non-assessable.

ROM	Pre-treatment	Pre-treatment		Post-treatment	
Side	Right	Left		Left	
Hip	Active	Active	Passive	Active	Passive
Flexion	0-120°	0-25°	0-50°	0-80°	0-110°
Extension	0-30°	0-10°	0-10°	0-15°	0-20°
Abduction	0-45°	0-15°	0-20°	0-20°	0-35°
Knee flexion	0-135°	NA	NA	0-60°	0-110°
Plantar flexion	0-50°	0-5°	0-10°	0-20°	0-40°
Dorsi flexion	0-20°	0-5°	0-10°	0-15°	0-20°
Inversion	0-35°	NA	0-15°	0-25°	0-30°
Eversion	0-15°	NA	0-10°	0-10°	0-15°

**Table 3:** Manual muscle testing (MMT).

MMT	Pre-treatment		Post-treatment		
Muscles	Right	Left	Right		Left
Hip flexors	4/5	1/5	5/5		4/5
Hip extensors	4/5	1/5	5/5		4/5
Hip abductors	4/5	1/5	5/5		4/5
Knee flexors	3/5	1/5	5/5		3/5
Knee extensors	3/5	1/5	5/5		3/5
Dorsiflexors	4/5	1/5	5/5	3/5	
Plantar-flexors	4/5	1/5	5/5	3/5	

Physiotherapy intervention

**Figure 4:**
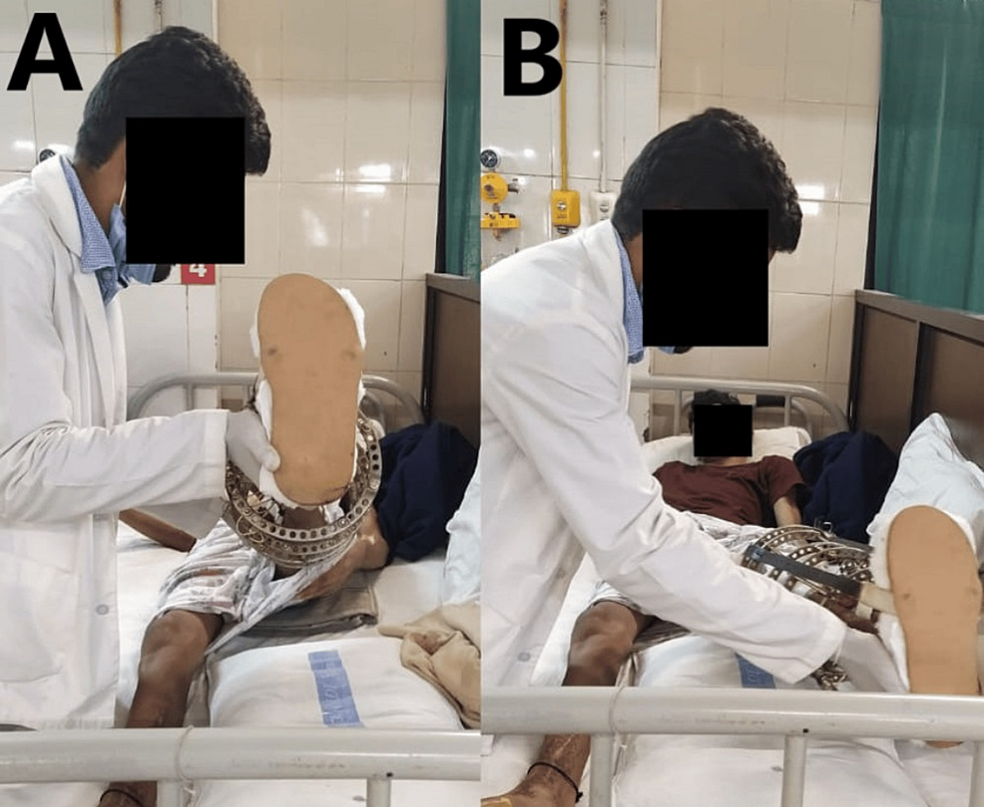
Passive range of motion in the left lower limb. A. Passive flexion of the left lower limb. B. Passive abduction of the left lower limb.

**Figure 5:**
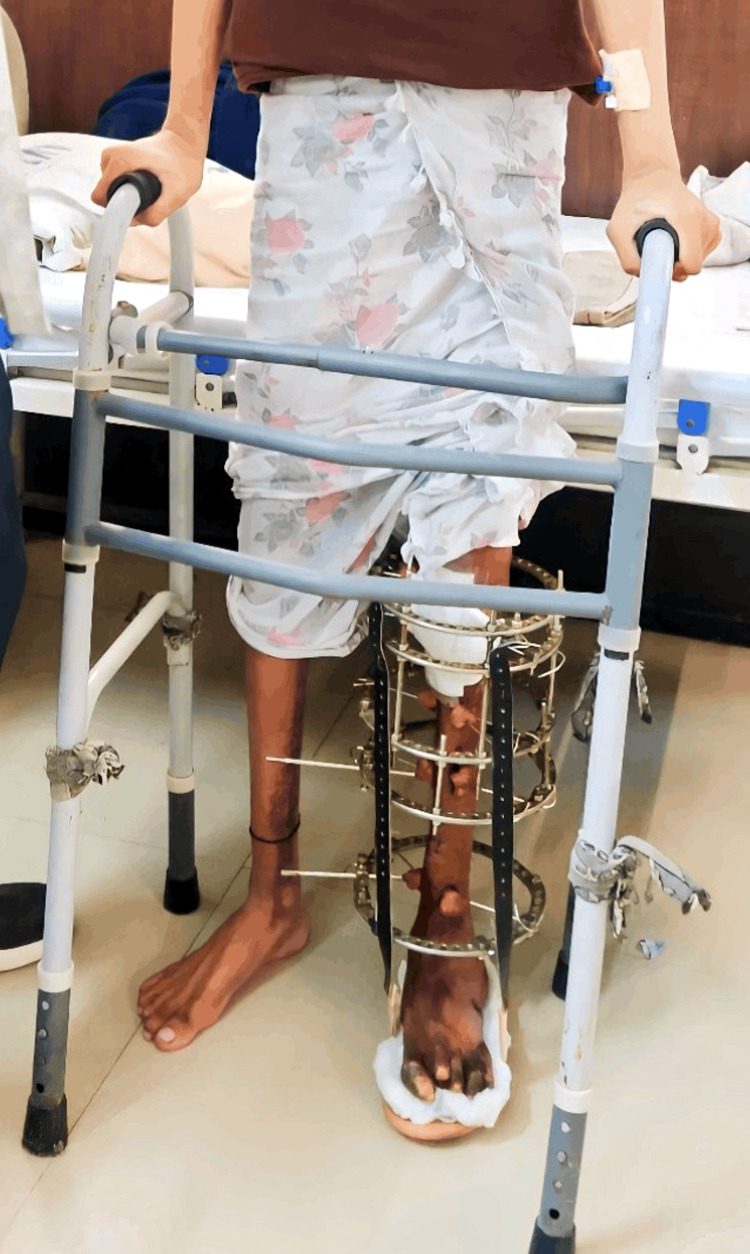
Full-weight bearing in standing with a walker.

The rehabilitation protocol for eight weeks of intervention is given below in Table 4. Improving the patient's quality of life (QOL) by helping him reach an ideal degree of independence in carrying out functional tasks and activities of daily living (ADLs) is the stated purpose of rehabilitation. Long-term objectives included promoting independent walking with walkers who could support their weight and required little outside assistance for daily tasks. The external fixator is a partial load-sparing device that can be used for provisional or definitive stabilization of fractures. The physiotherapy intervention given to the patient is shown below in Figure 4 with the patient performing passive movements by the therapist and in Figure 5, where the patient is standing in a full-weight-bearing position with a walker.

**Table 4:** Physiotherapy rehabilitation. reps: repetition; sec: seconds; ROM: range of motion; UL: upper limb; LL: lower limb; kg: kilogram; AAROM: active assisted range of motion; ADLs: activity of daily living; FEMS: functional electrical muscle stimulation.

Intervention	Goals	Phase 1 (inpatient day one-week two)	Phase 2 (inpatient week two-four)	Phase 3 (outpatient week four-six)	Phase 4 (outpatient week six-eight)
Patient education	The health benefits of exercise, the particulars of the fitness regimen, and the patient's family were all explained to them.	In all four phases, patient education was continuously provided.			
Positioning	To prevent from pressure sores	Patient is being positioned correctly and pressure sore-prone areas are being cushioned. Position shift occurring on a regular basis every two hours			
Active ankle-toe movements for right LL and passive ankle-toe movements for left LL due to motor impairment.	Promote proper blood circulation in the lower limbs.	Five reps x one set (three times/day) gravity eliminated position (supine)	Ten reps x one set (three times/day) gravity eliminated position (supine)	Ten reps x one set three times/day gravity assisted and gravity-resisted position (bedside sitting)	Ten reps x one set three times/day gravity-assisted and gravity-resisted position with minimal resistance by therapist’s hand (bedside sitting)
Isometric contraction for left quadriceps, hamstrings, and gluteal muscles	For strengthening the quadriceps, hamstrings, and gluteal muscles	Five reps x five-sec hold three times a day	Seven reps x five-sec hold three times a day	Seven reps x 10-sec hold three times a day	Ten reps x 10-sec hold three times a day
Deep breathing exercises	To prevent from chest complications and promote lung expansion	Five reps x set three times/day with 3-6-9 technique (three seconds inspiration, six seconds hold the breath, nine seconds expiration)	Ten reps x set three times/day with 3-6-9 technique	Ten reps x set three times/day with 3-6-9 technique	Ten reps x set three times/day with 3-6-9 technique
Actively resisted exercises for UL	To avoid contractures and to preserve both the strength and range of motion	Five reps x one set three times/day against gravity	Seven reps x one set three times/day - against gravity along with half kg weight cuff	Seven reps x one set three times/day - against gravity along with half kg weight cuff	Ten reps x one set three times/day - against gravity along with one kg weight cuff
Active straight leg raising for right leg and passive for left leg	Maintain hip and knee range and strengthen quadriceps	Five reps x five-second hold three times a day	Seven reps x five-second hold three times a day	Seven reps x ten-second hold three times a day	Ten reps x ten-second hold three times a day
AAROM exercises for affected LL hip abduction and adduction	Maintain hip abductors and adductor strength	Five reps x one set three times a day gravity eliminated position (supine)	Ten reps x one set three times a day gravity eliminated position (supine)	Ten reps x one set three times a day gravity-assisted and gravity-resisted position (side lying)	Ten reps x one set three times a day gravity assisted and gravity resisted position with minimal resistance by therapist’s hand (side lying)
Heel slides active to unaffected leg and passive to affected leg	Reduce stiffness, improve range, and maintain the strength of your hamstrings and quadriceps	Five reps x one set (three times/day)	Ten reps x one set (three times/day)	Ten reps x one set (three times/day) in the available range of motion	Ten reps x one set (three times/day) in the available range of motion
Unilateral pelvic bridging with weight-bearing to unaffected lower limb	To strengthen the hip and low back extensors and to facilitate pelvic motions	Five reps x five-second hold three times/day	Seven reps x five-second hold three times/day	Seven reps x ten-second hold three times/day	Ten reps x ten-second hold three times/day
Dynamic quads	Enhance strength of knee extensors	Five reps x one set three times/day actively for unaffected LL	Ten reps x one set three times/day actively for unaffected LL	Five reps x one set three times/day passively for affected lower limb and ten reps x one set three times/day actively for unaffected LL	Ten reps x one set three times/day passively for affected LL and ten reps x one set three times/day actively for unaffected LL
Sit-stand with walker	Enabling the patient to perform his ADLs independently	Five reps x one set (three times/day)	Five reps x one set (three times/day)	Ten reps x one set (three times/day)	Fifteen reps x one set (three times/day)
Weight-bearing with the walker to the left side	Promoting independent walking with walkers who could bear their own weight and require little outside assistance for daily tasks	Non-weight bearing	Toe touch weight-bearing	Partial-weight bearing	Full-weight bearing
Ambulation with walker	To make the patient independent for his ADLs	One round of 50 meters three times/day	Two rounds of 70 meters three times/day	Two rounds of 100 meters three times/day	Three rounds of 100 meters three times/day
Various textures and features of sensory stimuli were used to apply the sensory re-education technique to the left foot. Applying FEMS to the selected muscles.	Activate the muscles in the left foot to improve motor functions and sensation.	Vibratory stimuli are stroked proximally to distally over the left foot's dorsum and plantar surface. Galvanic stimulation induces dorsiflexion.	Proximal to distal stimulation over dorsum and plantar aspect of the left foot using vibratory and tactile stimuli (both static and dynamic). Dorsiflexor activation by galvanic stimulation	Proximal to distal stimulation over dorsum and plantar aspect of the left foot using vibratory and tactile stimuli (both static and dynamic). Dorsiflexor activation by faradic stimulation	Proximal to distal stimulation over dorsum and plantar aspect of the left foot using vibratory and tactile stimuli (both static and dynamic). Dorsiflexor activation by faradic stimulation

Pre-intervention and post-intervention outcome measure scores are mentioned in Table 5.

**Table 5:** Outcome measures. NPRS: numerical pain rating scale; LEFS: lower extremity functional scale; FAAM: foot and ankle ability measures.

Outcome measures	Pre-intervention score	Post-intervention score
NPRS	8/10	4/10
LEFS	12/80	30/80
FAAM	20%	55%
Stanmore assessment questionnaire	56/100 (poor)	80/100 (good)

## Discussion

The case report describes the successful rehabilitation of an 18-year-old male with a compound grade 3c proximal tibia fracture treated with Ilizarov fixators, complicated by neurovascular deficit and foot drop on the left side. The patient also had a previous femur shaft fracture managed with implants. The rehabilitation protocol, consisting of four phases from inpatient to outpatient care, aimed to improve muscle strength, range of motion, and sensory impairment in the left lower limb to restore the patient's independence in daily activities. The effectiveness of the physiotherapy intervention is evident from the positive outcomes observed, such as enhanced scores on the Lower Extremity Functional Scale, Foot and Ankle Ability Measures, and Numerical Pain Rating Scale. Additionally, there was a notable improvement in the Stanmore assessment questionnaire, with the score shifting from "poor" to "good". This case highlights the importance of a comprehensive and multidisciplinary approach in managing complex lower limb fractures, with physiotherapy playing a key role in achieving positive functional outcomes and improving the patient's quality of life.

In the study of Daf et al. ROM, muscle strength, and the patient's general functional capacity are all improved with physical therapy application during the early stages of post-operative rehabilitation, which helps prevent complications related to immobility and allows for a prompt return to everyday life. Ilizarov fixation is used to surgically treat foot drop deformity and neurovascular impairment. In order to manage the patient with persistent osteomyelitis during the early stages of physiotherapy, the case study provides a structured approach [12]. Mundada et al. outline an extensive rehabilitation strategy considering individuals with chronic osteomyelitis treated with an Ilizarov ring fixator and sequestrectomy in this case report. The case study comes to the conclusion that the patient's functional independence and ambulation were enhanced by an interdisciplinary group that included a specific surgical approach and customized physiotherapy rehabilitation. As it facilitates a speedy and full recovery, this is important [13]. Shahade et al. used an Ilizarov external ring fixator in an operative case comprising a 10-month-old malunited supracondylar fracture of the femur, osteomyelitis, and a proximal tibia-fibula fracture with right-sided foot drop. The case study centers on an appropriate innovative application of physical therapy procedures [14]. Despite following an intense physical therapy program, patients cannot shift their body weight to the limb that has the Ilizarov equipment attached to it or split their body weight evenly between their two lower limbs [15].

The Ilizarov technique may be viewed as a beneficial and successful treatment for recurrent or disregarded abnormalities because it has demonstrated positive results [16]. Physiotherapists with varying levels of expertise in treating patients with Ilizarov fixators contributed their insights to the research [17]. While one may argue that these people require conventional, excellent musculoskeletal rehabilitation, the potential for related soft tissue issues creates a separate population that needs further consideration and research [18].

## Conclusions

In conclusion, for the 18-year-old male patient treated with Ilizarov fixation and a foot drop splint for a compound grade 3C tibia fracture, post-operative physical therapy has greatly improved outcomes. The rehabilitation approach was effective in pain reduction, improving range of motion, muscle strength, and reducing sensory impairment observed. The patient's Lower Extremity Functional Scale and Foot and Ankle Ability Measures scores improved, which is a sign that the physiotherapy technique was successful in helping the patient regain their independence in daily activities. The case highlights the importance of a multidisciplinary approach in managing such cases, with physiotherapy playing a pivotal role in achieving optimal recovery and independence in everyday activities for the patient.
